# A 5-year field study showed no apparent effect of the *Bt* transgenic 741 poplar on the arthropod community and soil bacterial diversity

**DOI:** 10.1038/s41598-018-20322-3

**Published:** 2018-01-31

**Authors:** Lihui Zuo, Runlei Yang, Zhixian Zhen, Junxia Liu, Lisha Huang, Minsheng Yang

**Affiliations:** 1 Institute of Forest Biotechnology, Forestry College, Agricultural University of Hebei, 071000 Baoding, PR China; 2 Hebei Key Laboratory for Tree Genetic Resources and Forest Protection, 071000 Baoding, PR China; 3 College of life science, Hebei University, 071000 Baoding, PR China; 4 BiomicsTech Co.L td, 100083 Beijing, PR China

## Abstract

China is currently the only country that has commercialized genetically engineered tree species, and this has attracted worldwide attention. As a perennial tree species, transgenic poplar has a long growth cycle and needs to be tested for long-term ecological risks. The main purpose of this study was to explore the ecological safety of perennial transgenic poplars in arthropod community, physical and chemical properties of soil, gene flow, and soil microbial diversity. The study found transgenic poplars could effectively inhibit the number of pests. Moreover, transgenic poplar 741 did not affect the stability of the arthropod community. Studies on the microbial diversity of poplar showed that transgenic poplars did not affect the physical and chemical properties of the soil and the soil microbial community structure. Furthermore, the microbial community structure was obviously affected by location and season. The results showed that a 5-year-old transgenic 741 poplar did not pose an ecological risk, and did not affect the microbial community structure or functional diversity. This study provides a reference for the ecological security evaluation of transgenic poplars, and provides a theoretical basis for promoting the commercialization of transgenic poplars.

## Introduction

Poplar (*Salicaceae populus*) is one of the most widely distributed and adaptable forest and tree species in the world, which has important economic and ecological value. Moreover, poplar is one of the most important pulp raw and industrial materials in the world^1^. Unfortunately, the insect resistance of poplar is poor. Due to the rapid growth of the poplar planting area, and most plantations are pure forests, poplars often have large areas infested with insect pests, especially Lepidoptera and Coleoptera pests. These pests affect plant growth, and can even cause plant death, resulting in huge economic losses^2^. To date, pest control has been difficult. Large-area chemical control is not only expensive, but can also cause damage to the ecological environment. Therefore, insect resistance has become the primary goal of poplar breeders. So far, sources of insect resistant poplar germplasm are relatively scarce, and the traditional methods of insect resistance breeding are time-consuming and have little effect. The emergence and development of transgenic technology has greatly accelerated the process of poplar insect resistance breeding. Transgenic technology can be used to achieve breeding goals by improving varieties in a short period of time. Now, researchers have discovered and isolated many insect resistant-related genes, including *Bt*, protease inhibitor (*PI*), *AaIT*, α-amylase inhibitor, lectin, and chitinases. Different insect resistant genes have different insecticidal ranges and effects. The *Bt* gene is the most widely used and effective insect resistance gene. So far, more than 130 transgenic *Bt* plants have entered the experimental and research stages. The *Bt* gene has become one of the most widely used insect resistance genes in the world.

*Populus trichocarpa* was the third plant to have its complete genome sequenced, which have much smaller genomes and easy to genetically manipulate. Moreover, poplar is the natural host of *Agrobacterium tumefaciens*; as such, it is easy to use *A*. *tumefaciens*-mediated genetic transformation. Therefore, it is considered a model plant for tree genetic engineering research^3^. In 1986, Parsons confirmed that poplar could be genetically transformed to express foreign genes^4^. Since then, poplar insect resistance transgenic research has made great achievements^5–8^. China has been in the forefront of research on *Bt* transgenic poplars. Wu^9^successfully transferred the *Bt* gene to European black poplar (*Populas nigra*) for the first time in 1991; this was the prelude to insect resistance transgenic research. In 1993, Tian transformed a specific *Bt* gene that is toxic to Lepidopteran insects into European black poplar^10^. In 2000, Zheng obtained the transgenic *Cry1Ac* gene 741 poplar^11^. In 2002, several new poplar varieties, including an insect resistant transgenic *P*. *nigra* variety, and the transgenic 741 poplar, were commercialized. Therefore, China has become the first and only country in the world to commercialize transgenic poplar. Since the 1990s, there has been a rapid increase in the number of reports on *Bt* transgenic poplars. The poplar clones involved in transgenic *Bt* mainly include *P*. *nigra*^12^, *P. deltoides*^13^, *P. euram ericana*^14^, NC5339 (*Populus alba* × *P*. *grandidentata*)^15^, NL-80106 (*Populus dehoides* × *Populus simonii*)^16^ and 741 poplar [*P*. *alba* × (*Populus davidiana* + *P*. *simonii*) × *Populus tomentosa*]^17^ and so on^17,18^. The emergence of transgenic poplar has effectively reduced the occurrence of insect pests and the use of chemical pesticides, which provides valuable germplasm resources for poplar insect resistance breeding.

Transgenic plants have brought great benefits and convenience to human beings^19^; however, the safety of transgenic plants has attracted more and more attention. The ecological security of transgenic plants has become a hot topic and a major obstacle to the promotion of transgenic plants. China is the only country that has commercialized genetically modified poplar species, causing widespread concern in the world. Therefore, it will be necessary to conduct research on the safety of transgenic trees. However, poplar is a perennial tree with a long growth cycle. Therefore, it will be necessary to perform a long-term ecological risk assessment of transgenic trees before entering the field trial and commercial production stage. In this study, using transgenic poplar as a test material, exogenous gene expression and gene flow in transgenic poplar were monitored for many years. Moreover, the microbial diversity of endophytic bacteria, rhizoplane, and rhizosphere soil from 5-year-old plants were monitored to determine the safety of transgenic poplars systematically. This study provides data for the biosafety evaluation of transgenic poplar. It can further improve the safety evaluation of transgenic poplar, and provide more detailed guidance and a theoretical basis for the commercialization of transgenic poplar.

## Materials and Methods

### Test materials

In this study, the genetic material was 741 poplar (triploid) [*Populus alba* L. × (*P*. *davidiana* Dode + *P*. *simonii* Carr.) × *P*. *tomentosa* Carr.] female plants. The *Cry1Ac* and *Cry3A* genes were used to construct an expression vector and transferred into 741 poplar clones by an *Agrobacterium*-mediated method. After a series of screenings, transgenic lines were obtained and named Pb29 and CC84, which were highly resistant to Lepidoptera and Coleoptera pests. The experimental forest is located in Tianjin, P.R. China. Using a randomized block design, the experimental forest was divided into three replicates, 25 strains per plot, with plants spaced 2 × 4 m apart with protective nets set up around the plants. The topography, physiognomy, soil, air temperature, vegetation, cultivation management, and other natural conditions were consistent. Human management was consistent and no pesticides were used during the investigation.

### Test method

#### Foreign genes and gene flow detection

**Figure 1:**
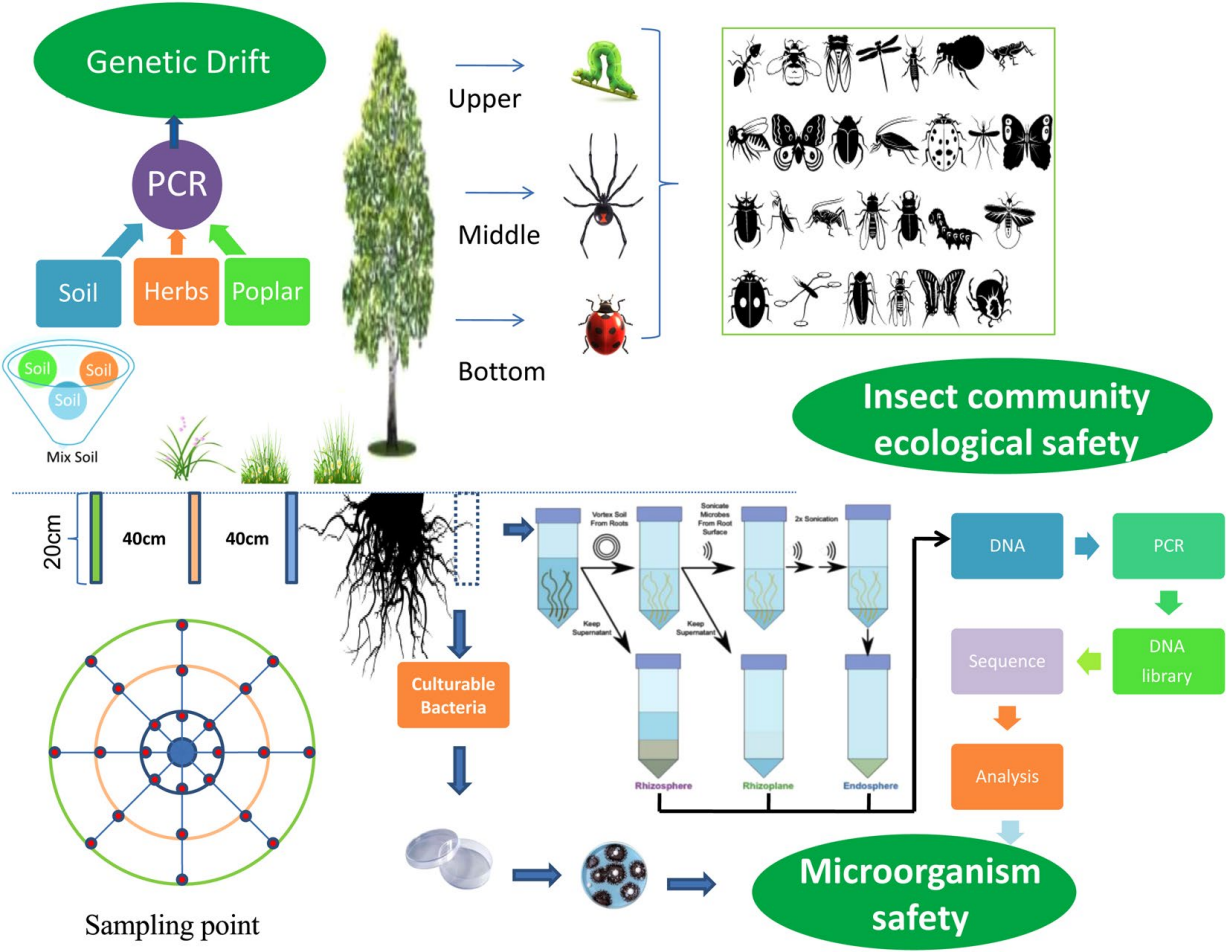
Test flow.

The undergrowth plants were sampled using a random sampling method. The leaves from all plants were collected from randomly selected 1 × 1 m quadrants in each plot. The leaves were taken back to the laboratory at low temperature in three repetitions. Similarly, three poplar trees were randomly selected in each plot, and a sampler was used to collect 20 cm of soil at different locations (East, West, South, North, Southeast, Northeast, Southwest, and Northwest) at different distances (40, 80, and 160 cm; avoiding plant roots) from the tree. A total of 24 sampling sites and mixed samples (Fig. 1) were taken back to the laboratory at low temperature for PCR detection of soil physicochemical properties and gene flow. The cetyl trimethylammonium bromide (CTAB) method was used to extract the total DNA from all poplar and understory vegetation samples. The DNA was diluted to 100 ng/μL for PCR detection of the target genes. Primers were designed according to the sequence of the target fragments. The sequences of the *Cry1Ac*, *Cry3A* and *NPTII* gene primers were: *Cry1Ac*-F: 5′-CAACCCGAACATCAACGAAT-3′, *Cry1Ac*-R: 5′-GCCAATAAGCCTAGTTAAATCA-3′; *Cry3*-F: 5′-TGGCCAAGCGAGGACCCCTGGAAG-3′, and *Cry3*-R: 5′-ACCGTCTCTGGTAAGCTCGGTCTT-3; *NPTII*-F: 5′-ATCTCCTgTCATCTCACCTTgCTCCT-3′, *NPTII*-R: 5′-TCAGAAGAACTCGTCAAGAAG-3′. PCR reactions (20 μL) were prepared using 12.8 μL deionized water, 2.0 μL 10 × PCR buffer (Tris-HCl pH 8.3, 500 mM KCl, and 15 mM MgCl_2_), 2.0 μL dNTPs (2.5 mM), 1.0 μL primer (10 mmol/L), 1.0 μL DNA, and 0.2 μL Taq enzyme (5 U/μL). The PCR program was as follows: pre-denaturation at 94 °C for 4 min; 30 cycles of denaturation at 95 °C for 1 min, annealing at 55 °C for 1 min, and 1 min of extension at 72 °C; followed by a final extension at 72 °C for 7 min. The PCR products were detected by 1% agarose gel electrophoresis.

#### Arthropod community analysis

From 2012 to 2015, the numbers of arthropods in the test forest were determined during the growing season (May to October). Using the random sampling method, three trees were randomly sampled from each plot, and all arthropods on the ground and branches were surveyed in detail. Each tree was divided into three layers, each of which was divided into four directions: East, South, West, and North. The numbers of arthopods were counted on a total of 12 branches. The arthropod community in the surface layer was investigated using the random quadrat method: three quadrats were selected randomly in each plot, and the insects in the quadrat were investigated separately.

#### Physical and chemical properties of the soil

Soil samples were air-dried and sieved through a 2-mm grid. Three rhizosphere soil samples (2 mm fractions) from each plant were analyzed for their pH, organic matter (OM) content, available nitrogen (AHN), available phosphorus (AP), and available potassium (AK) according to the standard of LY/T 1239–1999, LY/T 1237–1999, LY/T 1232–2015, LY/T 1234–2015, and LY/T 1228–2015. The soil pH was measured in a 1:2 (w/v) soil to water suspension using a pH meter. The OM content was determined by the Walkley–Black acid digestion method. AP (extracted with 0.03 M NH_4_F-0.02 M HCl) was measured with molybdenum blue colorimetry. AK was measured with an ammonium acetate method using a flame photometer, and AHN was measured with the alkaline hydrolysis diffusion method.

#### Detection of endophytic bacteria and soil microbial diversity

The soil environment of plant roots can be divided into three parts: bulk, rhizosphere, and rhizoplane. Bulk soil is the loose soil around the root system, rhizosphere soil is the soil in the root table 3–5 mm distance, and rhizoplane soil is the soil in the root table 0–2 mm distance. As shown in the flowchart in Fig. 1, samples were taken at different distances from the trunk and at a depth of 20 cm, avoiding roots and damaged fibrils, to expose the root. To sample rhizosphere soil, select roots with the same weight (5 g) were put in a erlenmeyer flask filled with 50 mL sterile water and placed on a shaking table for 15 min to obtain a rhizosphere soil suspension. Next, the root system was dried with sterile filter paper, weighed, and ground. Sterile water was added and the suspension was placed on a shaking table for 15 min to obtain a rhizoplane soil suspension. The suspension was diluted 10^5^ times, and cultured on beef extract peptone solid medium for 2 days at 37 °C. The number of bacteria (colony forming units [CFU]) was counted using the following formula: CFU = N*M/[L*(1-P)]. N: colony number; M: dilution times; L: inoculum size; and P: soil moisture content.

#### Diversity analysis of endophytic bacteria and soil bacteria in plants

As a large proportion of soil bacteria cannot be cultured, to accurately determine the effect of transgenic poplar on the soil bacterial community, we used high-throughput sequencing methods to identify bacteria present in different seasons (June, August, and October) at different sites (endophytic bacteria, rhizosphere, and rhizoplane) using the same sampling method as previously described. The total DNA from all samples in different seasons was extracted. The primers were synthesized according to the variable region of V3–V4 (F: 5′-CCTAYGGGRBGCASCAG-3′, R: 5′-GGACTACNNGGGTATCTAAT-3′) in the hypervariable region of 16 S rDNA and an adaptor sequence was added to the end of the primer. PCR amplification was performed, and the product was purified, quantified, and homogenized to create a sequence library. The completed library was tested using a library quality test. Then HiSeq. 2500 was used for high-throughput sequencing.

### Statistics and Analysis

The soil physicochemical properties and arthropod communities from different samples were statistically analyzed with SPSS17 software. The original data were spliced, and the spliced sequences were mass filtered to remove chimeras and obtain high-quality Tags sequences. The sequences were clustered at 97% level of similarity, and 0.005% of all sequences were sequenced as the threshold to filter operational taxonomic units (OTUs) by QIIME (version 1.8.0) software^20^. According to the Silva database, the bacterial community was annotated with RDP Classifier software, and the confidence threshold was 0.8. The alpha and beta diversity of different samples was analyzed by Mothur^21,22^ software (version 1.30), and principal component analysis (PCA)^23^ was performed using R software. STAMP software^24^ was used to perform the *t*-test to identify different species among different groups, and PICRUSt software^25^ was used to predict the function of the species. Linear discriminant analysis effect size (LEfSe) software^26^ (strictly screened tr 1, linear discriminant analysis [LDA] score 4) was used to determine if biomarker levels in different leaves were statistically different. A taxonomic dendrogram of bacteria was analyzed using MEGAN5 (http://ab.inf.uni-tuebingen.de/software/megan5/).

## Results and analysis

### Foreign genes and gene flow detection

**Figure 2:**
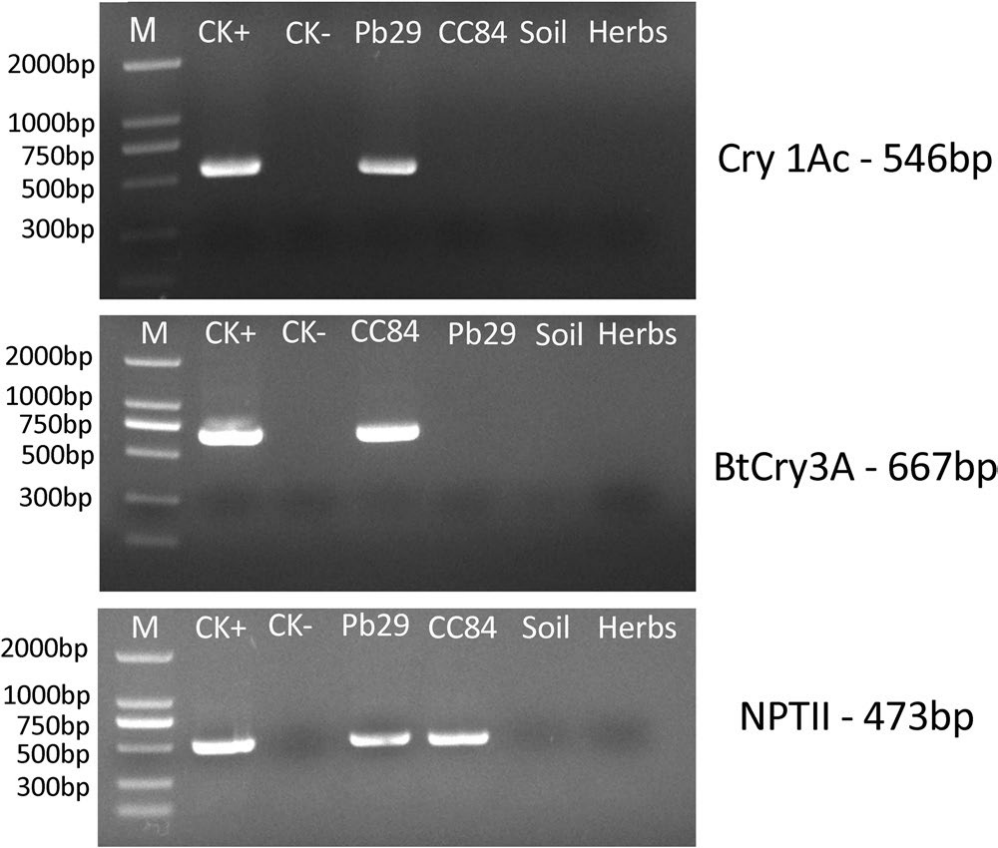
PCR detection of exogenous genes. Note: The full-length gels are presented in Supplementary Figure 11.

For five consecutive years, PCR was used to detect foreign genes. The *Cry1Ac*, *Cry3A*, and *NPTII* genes of transgenic poplar were detected and no gene loss occurred (Fig. 2). The *Bt* gene was not detected in non-transgenic 741 poplar, understory vegetation, and soil. This indicated that the transgenic Pb29 and CC84 poplars were safe, and there was no gene flow.

### Effects of the transgene on the arthropod community

**Figure 3:**
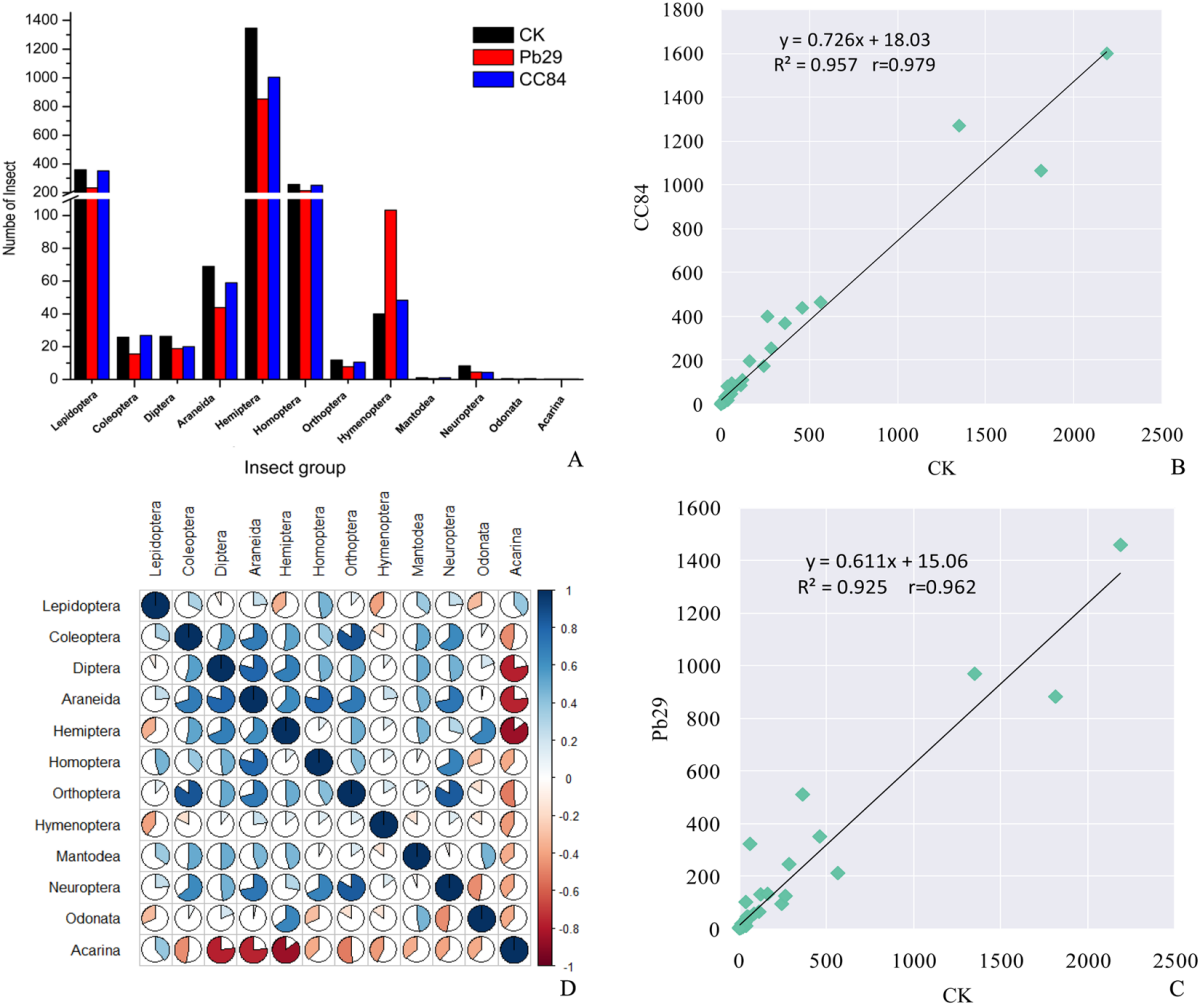
The number of species and individual of arthropod community.

The arthropod communities in the experimental forest were counted for four consecutive years. A total of 21,662 insects from 12 orders were recorded in 4 years (Fig. 3A). The arthropod community in transgenic 741 poplar was similar to that of control 741 poplar in structure and composition. The correlation coefficients were 0.962 and 0.979 (a very significant correlation level), respectively (Fig. 3B,C). The numbers of insects in the arthropod communities were investigated for four consecutive years and the results showed that the dominant species in all poplars was Hemiptera, followed by Lepidoptera, Homoptera, Araneida, and Diptera insects, while the numbers of Mantodea, Neuroptera, Odonata, and Acarina were relatively low. The main pests of poplar (primarily Lepidoptera) were obviously inhibited in the transgenic poplar; however, the number of Coleoptera pests was generally low, and the inhibitory effect was not obvious. At the same time, there was little influence on the number of natural enemies and neutral insects. This indicated that transgenic 741 poplar did not affect the insect ecological community. Therefore, transgenic poplar 741 is an ecologically friendly species and does not pose an ecological risk. Moreover, there was significant correlation between the numbers of insect communities in different types (Fig. 3D). A correlation analysis of different insect numbers showed that the correlation coefficients of different insects varied from −0.857 to 0.853. In relation to the major pests (Lepidoptera and Coleoptera), the number of Lepidoptera insects was positively correlated with Homoptera (mainly aphids) and natural enemy insects (Araneae, Diptera, and Mantis; not significant relative levels). Similarly, the number of Coleoptera was positively correlated with Diptera (neutral), Araneida (natural enemy), Hemiptera (vermin), Homoptera (vermin), and Orthoptera (neutral). The highest correlation was with Orthoptera (0.853), followed by Araneida (0.698; natural enemy), and Neuroptera (0.634; neutral). These results showed that an increase in the number of insect pests in poplar led to an increase in the number of natural enemies. At the same time, the insect ecological community is very complex and can influence each other and form a complex food chain. To our relief, the transgenic 741 poplar did not adversely affect the structure of the arthropod communities.

### Physical and chemical properties of the soil

Soil is the carrier for plant growth and development. It is an important place for the exchange of substances and energy in the ecosystem. The physical and chemical properties of soil are important indicators of soil quality. The safety and stability of soil physical and chemical properties directly affect plant growth. Therefore, we analyzed the physical and chemical properties of soil to explore if long-term growth of transgenic poplar could affect the physical and chemical properties of the soil. The soil analysis showed that there were no significant differences in hydrolyzable nitrogen, organic matter, available potassium, and available phosphorus content (Table 1). Only the soil pH was different; however, the change in soil pH was greatly influenced by the season. In June, the soil pH values of CK were significantly higher than those of Pb29 and CC84. However, there was no significant difference in soil pH between CK and Pb29 in August. Moreover, the soil pH of CK was not significantly different compared with Pb29 and CC84 in October. This showed that the change in pH in transgenic plant soil was not caused by transgenic events, but rather seasonal (or environmental) conditions. The above results indicate that the transgenic poplar did not affect the safety and stability of the physical and chemical properties of the soil. Furthermore, long-term growth of transgenic poplar did not have adverse effect on the physical and chemical properties of the soil. Therefore, transgenic 741 poplar is an environmentally friendly species.

**Table 1:** Determination of physical and chemical properties of the soil.

Sampe time	Clone	pH	AHN (mg/kg)	OM (g/kg)	AK (mg/kg)	AP (mg/kg)
**6**	CK	7.34 ± 0.03^a^	55.5 ± 6.2^a^	20.5 ± 3.1^a^	328 ± 17^a^	5.0 ± 0.6^a^
	Pb29	7.25 ± 0.03^b^	56.8 ± 6.0^a^	18.9 ± 1.0^a^	270 ± 20^a^	7.0 ± 0.8^a^
	CC84	7.27 ± 0.03^b^	61.7 ± 3.9^a^	22.2 ± 3.3^a^	287 ± 28^a^	6.0 ± 0.5^a^
**8**	CK	7.24 ± 0.03^b^	55.8 ± 8.3^a^	20.3 ± 2.2^a^	341 ± 54^a^	5.4 ± 1.6^a^
	Pb29	7.24 ± 0.02^b^	56.5 ± 2.4^a^	19.6 ± 1.5^a^	303 ± 42^a^	6.1 ± 0.6^a^
	CC84	7.34 ± 0.04^a^	65.6 ± 7.9^a^	22.5 ± 0.6^a^	286 ± 47^a^	5.0 ± 0.2^a^
**10**	CK	7.28 ± 0.05^ab^	65.3 ± 12.2^a^	21.4 ± 4.0^a^	309 ± 68^a^	6.1 ± 4.1^a^
	Pb29	7.35 ± 0.05^a^	54.9 ± 7.0^a^	20.3 ± 1.2^a^	320 ± 30^a^	7.0 ± 3.0^a^
	CC84	7.25 ± 0.03^b^	65.4 ± 1.3^a^	18.7 ± 4.5^a^	315 ± 21^a^	4.8 ± 0.9^a^

The English in this document has been checked by at least two professional editors, both native speakers of English. For a certificate, please see: http://www.textcheck.com/certificate/y4DAqg.

### Continuous monitoring of culturable bacteria for 5 years

**Figure 4:**
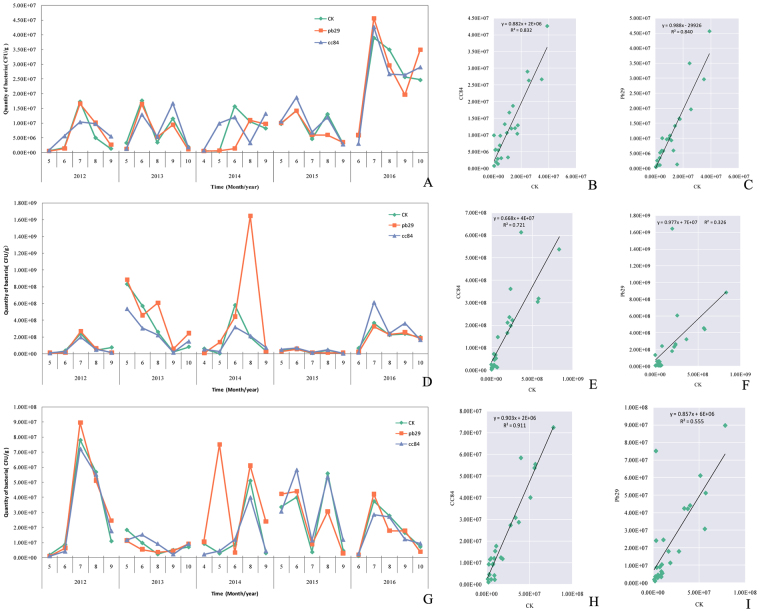
Dynamic changes in the number of culturable bacteria.

The bulk soil is far away from the plant root, and is usually not affected by plant root system infiltration. The nutrients and microorganisms in the bulk soil were relatively low. The culturable bacteria isolated from the bulk soil were monitored for 5 consecutive years. The number of bacteria isolated from the bulk soil showed an obvious seasonal pattern, but the rule was different in different years. In 2012, the number of bacteria varied with a unimodal pattern. Furthermore, the number of bacteria was low (May to June) and showed an upward trend that reached a peak in July and then decreased. However, the number of bacteria showed an “M” shape change in 2013 and 2015. In these years, the number of bacteria had two obvious peaks, indicating that the bacterial activity was strong. In contrast, the number of bacteria in 2016 was significantly higher than in other years, and showed a unimodal pattern (Fig. 4A). Furthermore, correlation analyses between the transgenic lines and non-transgenic strains for 5 years showed that both lines reached a very significant level (Fig. 4B,C). The results showed that the transgenic poplar did not affect the number of bacteria isolated from the root soil.

We also monitored all poplar rhizosphere bacteria for 5 consecutive years (Fig. 4D). The number of culturable bacteria in rhizosphere soil fluctuated greatly. The fluctuations had obvious seasonality and the number of bacteria varied every year. Overall, the number of bacterial communities in rhizosphere soil between transgenic and non-transgenic poplars was basically the same (expept Pb29-August). Among them, CC84, Pb29, and CK reached a very significant correlation level, indicating that the transgene did not affect the culturable bacteria in rhizosphere soil. In contrast, the composition of bacteria in the rhizosphere soil of CC84 was closer to that of CK (r = 0.849). On the other hand, the fluctuations from Pb29 were relatively large and the correlation coefficient was only 0.571 (Fig. 4E,F).

The rhizoplane soil is closely related to the plant root system, which is closely related to plant growth and metabolism. Similar to the results for bulk and rhizosphere soil, the number of bacteria in rhizoplane soil also had obvious seasonality, with larger fluctuations occurring during the growing season. Five years of monitoring data showed that the transgenic poplars did not affect the number of rhizoplane soil bacteria (except in May 2014 for Pb29) (Fig. 4G). The composition of bacteria in rhizoplane soil greatly reflects the physiological metabolic activity of the plant. In this study, the correlation coefficient between CC84 and CK was 0.955, while the correlation coefficient between Pb29 and CK was only 0.723 (Fig. 4H,I). These results demonstrate that the transgenic events did not affect the normal physiological metabolism of CC84, while the transgenic events caused disturbances and influenced the normal physiological metabolism of the Pb29 strain, and then affected the composition and quantity of root soil bacteria. Overall, 5 years of monitoring showed that transgenic poplar 741 did not affect the number of culturable bacteria in the soil; that is, the transgenic poplar 741 did not cause harm to the soil microbial environment.

### Soil microbial diversity

Due to the large proportion of soil bacteria that is unculturable, we also used high-throughput sequencing methods to examine the bacteria in rhizosphere, rhizoplane, and root endophyte at different times in 2016. After quality control analyses of the raw data, a total of 3,415,704 clean PE [paired-end reads (removed index, barcode, and linker sequences)] were obtained. There were 3,339,307 raw tags, 3,199,484 clean tags, and 2,665,220 effective tags with an average length of 449 base pairs. The GC content was about 55%, and the Q20 (%) and Q30 (%) of all test samples were above 95% and 92%, respectively. This showed that the sequencing quality was high and met the requirements for subsequent tests.

The total bacterial counts from transgenic and non-transgenic poplar were similar. The bacterial counts from non-transgenic poplars were slightly lower than that of transgenic plants, indicating that the transgenic poplars had little influence on the number of bacteria. The number of bacteria in rhizosphere and rhizoplane soil was similar in different growing seasons, and the fluctuations were small. The number of endophytic bacteria was significantly lower than the number of rhizosphere and rhizoplane soil bacteria. The number of endophytic bacteria varied seasonally. The number of Pb29 and CC84 bacteria showed a gradual decline in the growing season and exhibited large fluctuations. In June and August, there were no differences in the number of endophytic bacteria between transgenic and non-transgenic lines (CC84 had slightly higher bacterial counts than CK, and Pb29 had slightly lower counts than CK). However, the gap between transgenic lines and CK increased in October. The number of endophytic bacteria in transgenic strains showed a downward trend, while that of non-transgenic lines showed an upward trend (Fig. S1). This shows that the growth of endophytic bacteria was inhibited in the transgenic lines, but the number of soil bacteria in rhizosphere and rhizoplane soil was unaffected.

**Figure 5:**
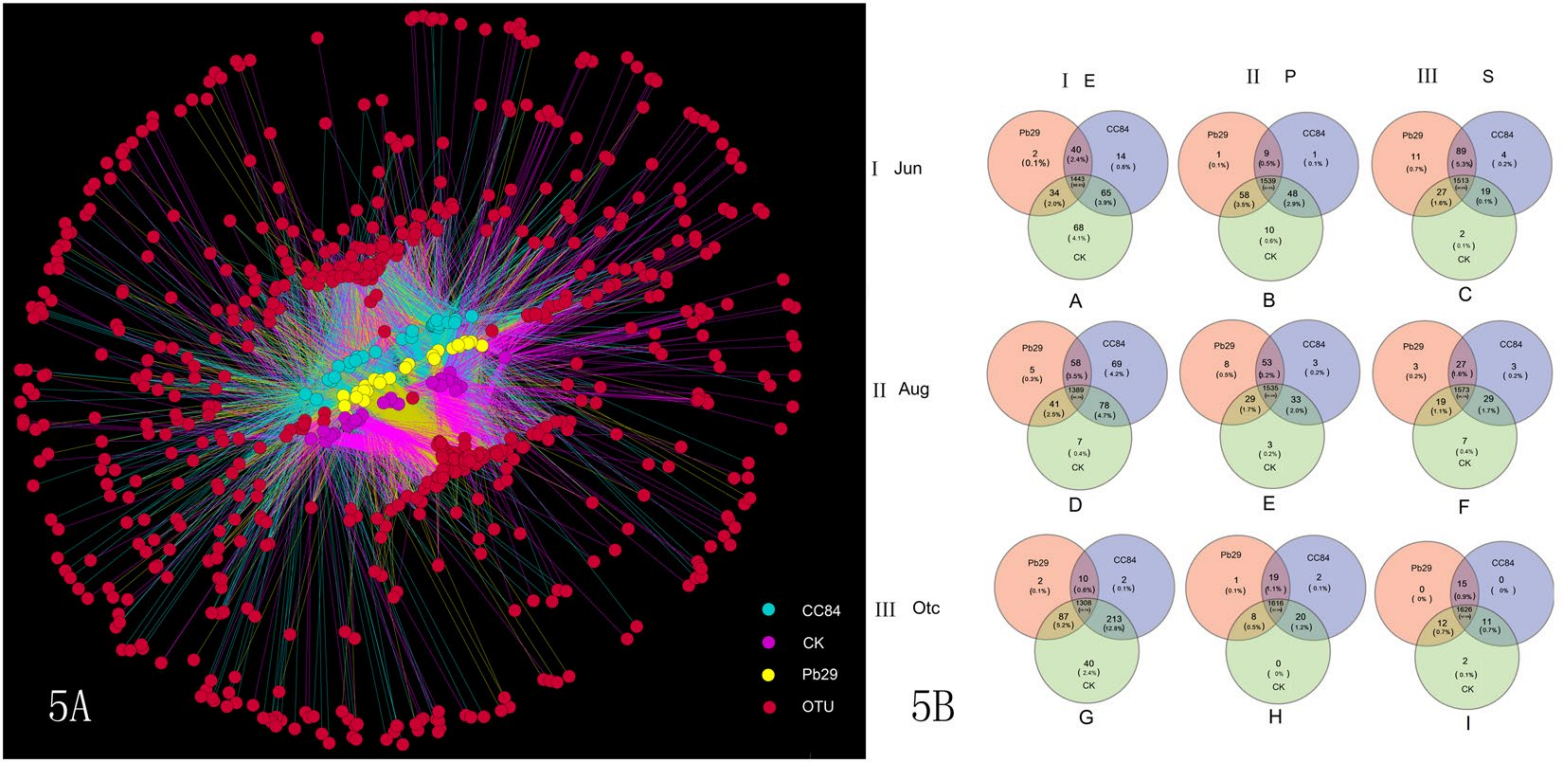
OUT netWork and OTU Venn diagram of different samples.

The OUT netWork and Venn diagram intuitively illustrates the common and unique OTUs. In combination with the bacterial species represented by OTUs, core microorganisms in different environments can be identified. OUT netWork analysis of all OTUs (97% similarity levels, absolute abundance >20) showed that the transgenic poplar had a great influence on endophytic bacteria, but little influence on the rhizosphere and rhizoplane soil bacterial community (Fig. 5A). Venn analysis (Fig. 5B) showed that the endophytic bacteria shared by transgenic and non-transgenic poplars varied from 84.3–86.6% (lowest in October and highest in August). This may have been due to the overexpression of exogenous genes, which affects the normal metabolism of plants and the composition of endophytes through their own metabolism. In contrast, transgenes did not affect the bacterial communities in rhizosphere and rhizoplane soil. Among transgenic and non-transgenic poplars, there were 92.2–97.0% (lowest in August and highest in October) and 90.9–97.6% (lowest in June and highest in October) common bacteria in rhizosphere and rhizoplane soil, respectively.

### Species annotation and relative abundance

All of the OTU sequences were compared with a microbial reference database to obtain corresponding species classification information. The results showed that there was almost no difference between bacterial abundance in transgenic and control poplars, and the correlation between bacterial community composition was 0.9997 and 0.9992, respectively (Fig. S2). This indicates that the transgene had no effect on soil bacterial diversity. At the phylum level, Proteobacteria was the dominant phylum with an average ratio in each sample of 56.2%, followed by Acidobacteria (11.9%), Bacteroidetes (10.7%), Actinobacteria (9.1%), Candidate_division_TM7 (3.6%), Chloroflexi (2.8%), Planctomycetes (1.5%), Verrucomicrobia (1.5%), and Gemmatimonadetes (1.0%). However, there were significant differences in bacterial community composition at different sampling sites. Among them, the differences were relatively large in Actinobacteria (E-13.81%, P-6.80%, and S-6.54%), Acidobacteria (E-9.52%, P-10.60%, and S-16.04%), Bacteroidetes (E-8.21%, P-12.52%, and S-11.17%), and Candidate division TM7 (E-4.21%, P-4.19%, and S-2.54%). The distribution showed that these strains had obvious location preferences. Compared with the different sampling sites, the composition of the bacterial community at different times was relatively small (Fig. S3); the largest fluctuations were from Proteobacteria (June, 58.70%; August, 53.17%; and October, 55.63%), Bacteroidetes (June, 9.77%; August, 12.01%; and October, 10.96%) and Acidobacteria (June, 11.25%; August, 12.76%; and October, 12.69%). This indicated that seasonal change had more influence on the bacteria.

The heat map of 19 main bacterial species (phylum level) was used to show the relationship between bacterial composition and relative abundance in transgenic and non-transgenic lines. The results showed that the transgenic lines were not significantly different from the non-transgenic lines (Fig. S4). This indicated that the transgenic poplars did not affect the diversity of microbial communities. However, there were obvious differences in the diversity of bacteria between different positions. Different parts of the bacteria form different branches, respectively. Moreover, the abundance of bacteria in 13 phyla (SHA−109, Verrucomicrobia, TM6, Acidobacteria, Planctomycetes, Chloroflexi, JL−ETNP−Z39, Chlorobi, Gemmatimonadetes, Nitrospirae, Bacteroidetes, Candidate_division_WS3, and Firmicutes) increased gradually from endophyte to rhizosphere and rhizoplane soil, while the bacteria in the remaining six phyla were insensitive to position.

### Alpha diversity

The alpha diversity analysis (Fig. S5) indicated that there was no significant difference between the transgenic and non-transgenic lines in the richness index (Ace and Chao1) and diversity index (Simpson and Shannon). Moreover, the richness and diversity indices of the non-transgenic lines were slightly higher than that of the transgenic plants. This showed that the transgenic plants had a slight influence on microbial diversity, but the effect was weak. Further analysis showed that the majority of these differences were due to sampling location and sampling time. As far as sampling location was concerned, the richness index (Ace and Chao1) of rhizosphere soil samples was the highest, followed by rhizoplane and endophyte soil. The diversity of bacteria in rhizosphere soil was higher than that in rhizoplane and endophytic soil. A comparison of samples at different sampling times showed that different seasons had little influence on the bacterial richness index. The bacterial richness index was higher in June and October, while the bacterial diversity index was highest in August. Overall, the number of species in the rhizosphere bacterial community was lowest, whereas the number of bacteria in rhizosphere and rhizoplane soil was relatively high. Moreover, the bacterial community diversity showed an increasing trend from endophytic to rhizoplane and rhizosphere soil.

### Beta diversity

**Figure 6:**
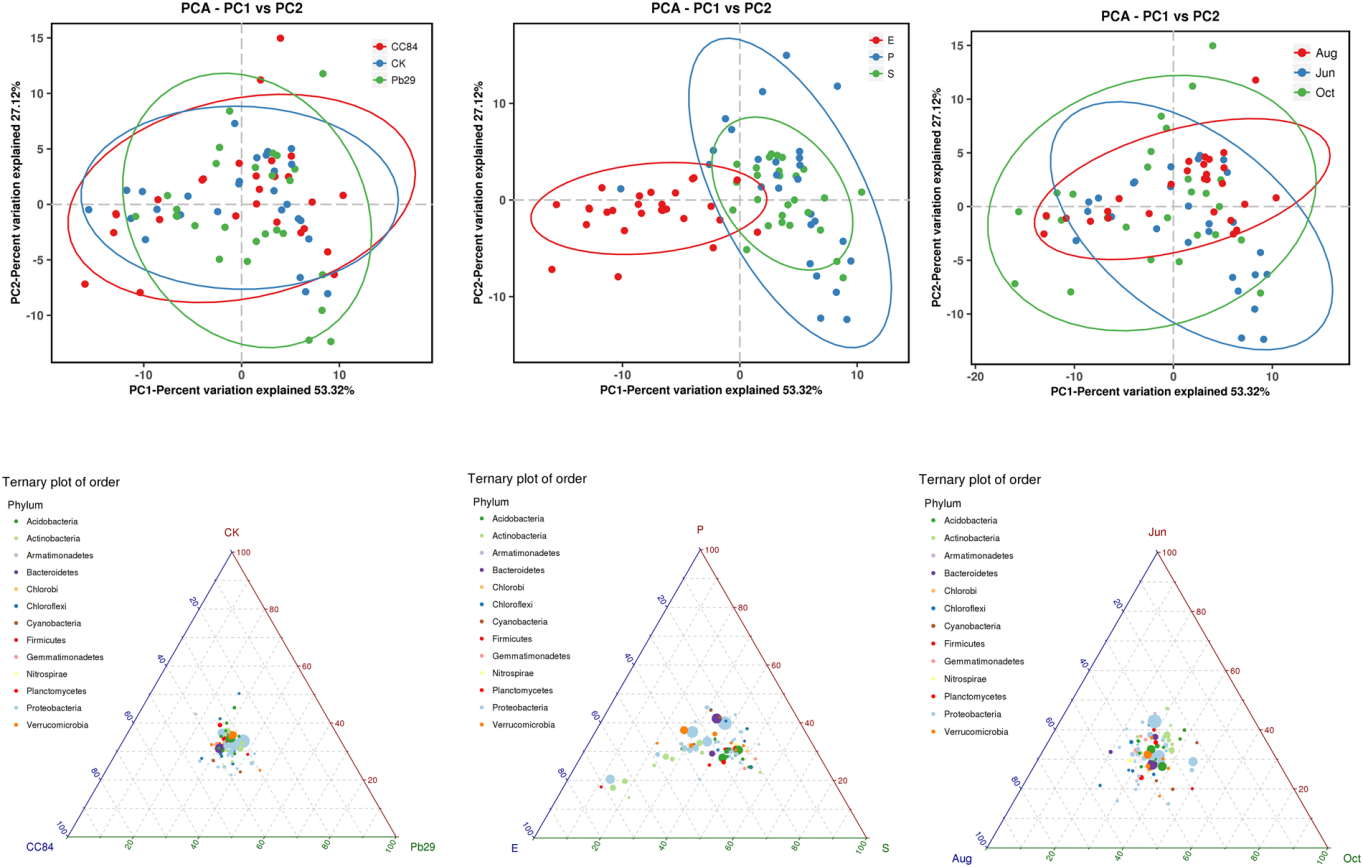
PCA and Ternary Plot result of different samples.

A PCA of all samples showed that the cumulative contribution rate of the variance of the first two principal components was 80.44%, which could explain the difference in bacterial community structure in most samples (Fig. 6). Most of the transgenic and non-transgenic lines overlapped and could not be separated. This indicated that the bacterial community composition of transgenic and non-transgenic lines was similar; that is, the transgenic poplar lines did not affect the bacterial community. There were obvious differences in bacterial community composition at different sites. The bacterial community composition in rhizoplane and rhizosphere soil was similar, mostly in the overlap region. However, the bacterial community composition from endophytic soil differed greatly from that of rhizoplane and rhizosphere soil, and separated into a single group, indicating that there were great differences in bacterial communities between locations. The vast majority of bacterial communities overlapped in different months. In addition, some of them showed obvious seasonality; that is, different growth stages influenced the community structure of bacteria. Similar results were obtained from a ternary diagram (phylum level) analysis; in other words, the similarity between microbial community structures was higher between strains, although there was a big difference between different parts of the bacterial community structure. Furthermore, sampling time had an impact on microbial community composition.

**Figure 7:**
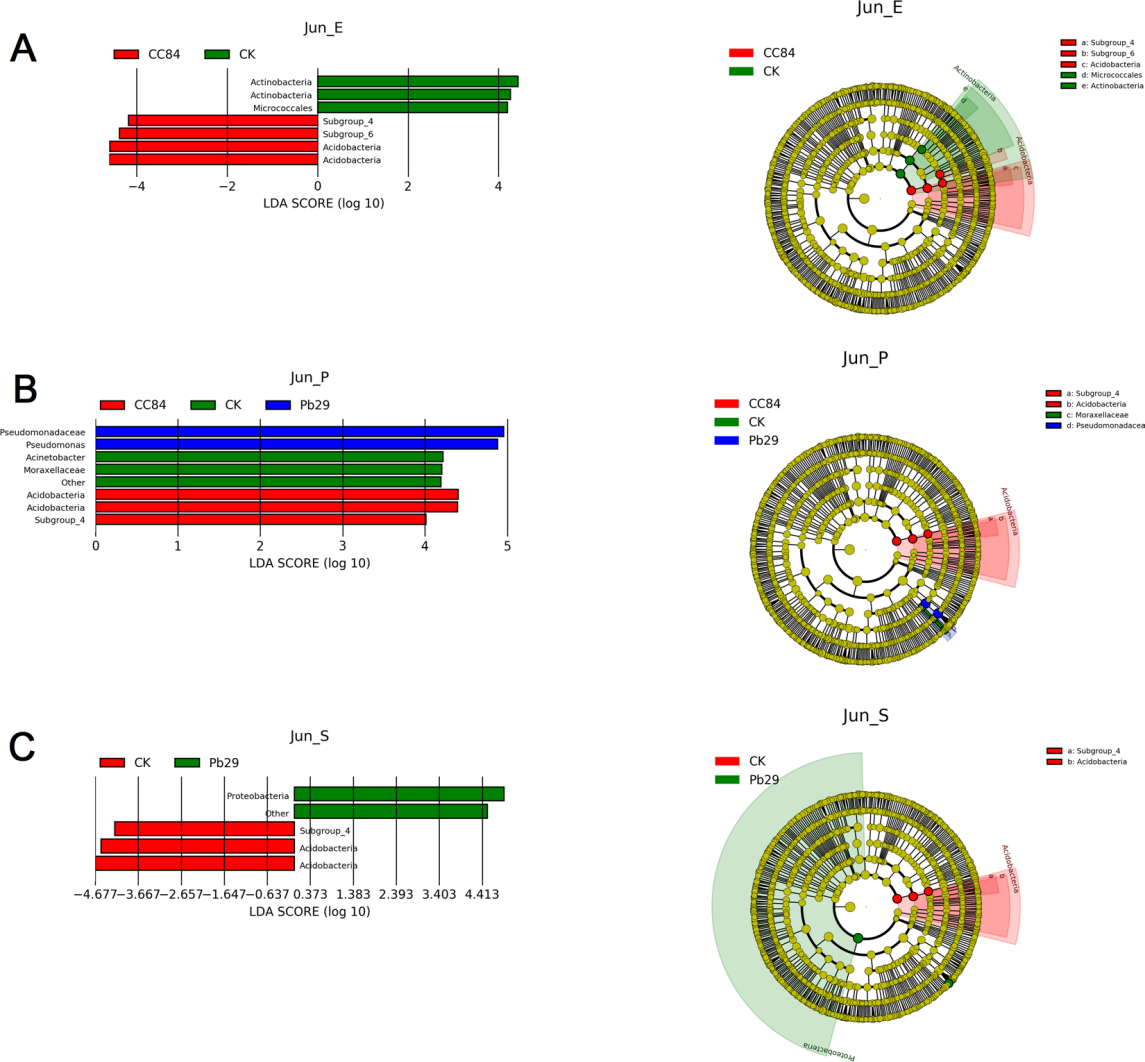
LDA score histogram and evolutionary branches of different samples.

The alpha and beta diversity analyses showed that transgenic poplars did not affect bacterial composition; however, the previous Venn diagrams showed that there were still a few specific OTUs among the different strains. Therefore, on the basis of annotation, we have performed a more rigorous LEfSe analysis. The difference between transgenic and control lines was highest in June, followed by August, but no difference was found in October (Fig. 7). Moreover, the bacterial communities at different locations also differed greatly. LEfSe analysis of endophytic bacteria in June showed that Micrococcales, Actinobacteria, and Actinobacteria were significantly different in CK. Among the CC84 endophytes, significant differences were found in subgroups 4 and 6 and Acidobacteria. In the rhizoplane soil samples of CK, Moraxelleceae and Acinetobacter were significantly different, while in CC84, the significant differences were found in Acidobacteria, Acidobacteria, and subgroup 4. Moreover, Pseudomonadaceae and Pseudomonas were significantly different groups in Pb29.

LEfSe analysis of samples in August showed that the significant endophytic bacteria groups were Rickettsiales and Mitochondria in CC84 (Figs S6, S7). In the samples of rhizoplane soil, the significant bacterial communities were subgroup 6 (CK) and Proteobacteria (CC84). There were no differences in bacterial communities between all samples of Pb29 compared with control group samples. Generally, there were a few differential bacteria among the three poplar lines in June. In August, only the CK and CC84 lines had differential bacterial species in endophytic and rhizoplane soil. However, there were no differences in bacterial species in all samples in October. These results showed that, although there was a small difference in bacterial diversity between transgenic and control poplars during growth; the difference was marked by seasonality, which is an environmental factor. That is to say, transgenic poplars did not affect the bacterial community.

### 16 S functional gene prediction analysis

**Figure 8:**
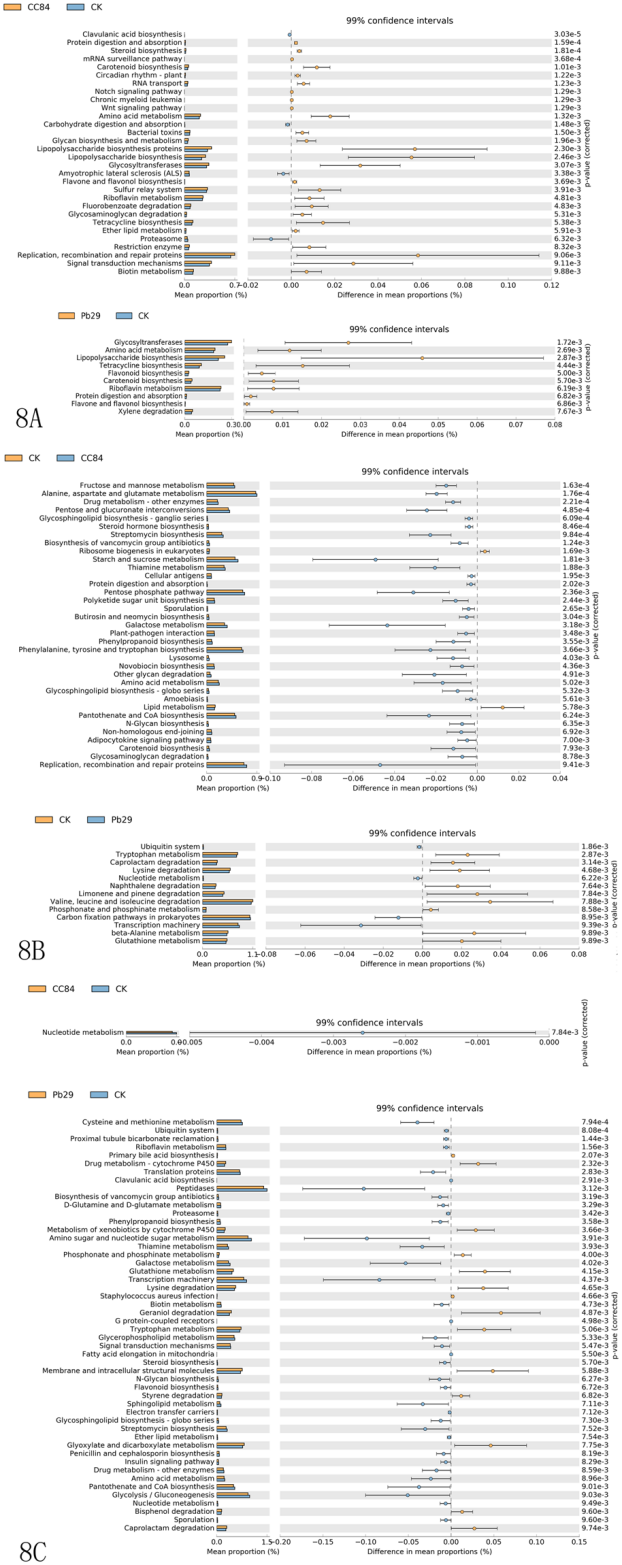
KEGG pathway T-test of different samples.

Using the Kyoto Encyclopedia of Genes and Genomes (KEGG) metabolic pathway analysis, we compared the differences and changes in metabolic pathways of functional genes in microbial communities between transgenic and non-transgenic poplar samples. The result showed that there were 30 metabolic pathways that were significantly different in endophytic bacteria from transgenic line CC84 than CK in June (Fig. 8A). Moreover, the abundance of pathway (26, 86.67%) related bacterial communities in CC84 was higher than that in CK. For example, proteins related to glycosyltransferases, lipopolysaccharide biosynthesis, lipopolysaccharide biosynthesis, signal transduction mechanisms and replication, recombination, and repair were all increased. Similarly, there were 10 pathways where the abundance of bacteria in the transgenic line Pb29 was higher than that of CK. These pathways were mainly related to xylene degradation, flavone and flavonol biosynthesis, protein digestion and absorption, riboflavin metabolism, and carotenoid and flavonoid biosynthesis. These results indicate that exogenous gene expression has an impact on plant metabolism and the composition of endophytes.

In rhizoplane soil, the bacteria that were significantly different in abundance were involved in 13 and 35 pathways, respectively (Fig. 8B). In rhizosphere soil, Pb29 greatly influenced the bacterial community, with 50 pathway changes in total, while only one change was identified in CC84 (Fig. 8C). However, this change was not absolute, and there was obvious seasonality. As time progressed, the difference between transgenic and control lines gradually decreased. For example, in August, there were no significant differences in rhizosphere soil between Pb29 and CC84 (Fig. S8–S10). In October, there were no significant differences in the bacterial community between CC84 endophyte and rhizoplane soil in CK. These results indicate that the changes in the microorganisms were not caused by transgenesis, but rather environmental changes.

## Discussion

Poplar is the most widely distributed and adaptable forest and tree species in the world, and has important economic and ecological value. However, during growth, poplars often suffer from insect pests, especially Lepidoptera and Coleoptera, which affect plant growth, and can even cause death, resulting in great economic losses. Pests are major problems that plague mankind. Chemical pesticides are expensive and broad spectrum; meaning non-target insects are also harmed, leading to disturbances in the ecological balance. Chemical pesticides also pollute the atmosphere, water bodies, soil, and crops. Residual pesticides migrate and transform among the elements of the environment, and are enriched by the food chain, which finally cause harm to organisms and human beings. The transgenic *Bt* poplar inhibited the occurrence of pests, decreased the use of pesticides, and protected and maintained the ecological environment. In recent years, the safety of transgenic plants has become the focus of human attention^27^. Regardless of whether transgenic plants have potential harm to the ecological environment, long-term monitoring is needed. In this study, transgenic poplar was used as the test object. The arthropod community, gene flow, and soil bacteria were tested for 5 consecutive years, and the safety of transgenic poplar was evaluated systematically and comprehensively.

### Effects of transgenic poplar on ecological environment

The limb fauna is a complex food web that contains a variety of phytophagous, natural enemy, and neutral insects. They interact with each other through nutrient flow and chemical communication in the food chain. The changes in species composition or quantity of a nutrient layer can be reflected in this complex network system, which can manifest as direct or indirect effects on other trophic layers through nutrient flow or information flow. The aim of engineering the transgenic *Bt* gene poplar was to inhibit target insects (mainly Lepidoptera and Coleoptera) and mitigate their harm to poplar. As an important component of arthropod communities, weather the decrease in the number of target insects could affect the stability of other insects, food chains, and even insect communities or not, long term observations are needed. Whether transgenic plants are harmful to limb fauna is the focus of scholars’ attention. Zhang studied the arthropod community from transgenic poplar (*P*. *alba* × *P*. *glandulosa*) carrying the *Bt-Cry3A* gene and found the *Bt-Cry3A* gene decreased damage caused by the target pest (*P*. *versicolora*), had no effects on a non-target pest (*C*. *anachoreta*), and generally did not have any significant negative effect on the poplar arthropod community^28^. Kim^29^ analyzed the arthropod community from transgenic *Cry1Ac1* cabbage, and found that the transgene reduced the number of target insects, had no effect on non-target insects, and posed no ecological risks. In this study, we monitored the arthropod communities of transgenic poplars for 5 consecutive years. Studies have shown that transgenic poplar effectively inhibits the number of target pests and has little effect on non-target insects. Moreover, the quantity of insects fluctuated greatly in different years. But on the whole, the arthropod communities of transgenic poplars and controls reached a very significant correlation level (r = 0.962 and r = 0.979, respectively). These results indicate that the transgenic *Bt* poplar did not affect the number of arthropods in the community. Therefore, the transgenic poplar did not pose a risk to the ecological environment.

Gene flow refers to the spontaneous transfer of a biological target gene towards nearby wild relatives, which lead to genetic changes within wild relatives of nearby species. Gene flow eventually leads to the formation of new species with dominant characteristics of target genes. Although pollen and seeds usually mediate gene flow, some gene flow is a result of asexual propagation, which would typically result in the long-term survival and the spread of plant residues to the soil or to other new plants. The transgenic material used in this study was 741 poplar (hybrid), which was formed by sexual hybridization breeding with the hybridized combination of *P*. *alba* L. × (*P*. *davidiana* Dode + *P*. *simonii* Carr.) × *P*. *tomenlosa* Carr. First of all, poplars are a dioecious tree species and only female trees were used in this study to prevent the possibility of gene flow by pollen. Secondly, the trees were triploid and the seeds were dry and highly sterile. We conducted sexual hybridization with the pollen of *P*. *tomentosa*, *P*. *alba* × *P*. *tomentosa*, and *Populus beijingensis* on 741 poplars for 3 years and found that the seeds were not normally developed. Anatomical experiments showed that the seeds stopped developing 2 weeks after pollination and finally failed. Therefore, the possibility of gene drift through seeds was almost zero. Finally, we tested the undergrowth vegetation and soil microorganisms for 5 years, but no target genes were detected. This indicated that the transgenic poplars were safe and there was no risk of gene flow.

### Effects of transgenic poplars on the bacterial community

The results showed that the number of microorganisms at different times and sampling sites varied greatly. The composition of microorganisms can reflect soil characteristics to some extent, and the composition of bacteria in the soil can be used as an indicator of the ecological condition of the soil. For example, there were higher utilization rate of Betaproteobacteria, Bacteroidetes, and Pseudomonas when the soil was rich in carbon, while the number of Acidobacteria rose when the soil was poor. As shown in this study, the proportion of Acidobacteria and Bacteroidetes in August samples was higher than that in June and October; this may have been due to the relatively higher abundance of nutrients available in the environment in August. In addition, the composition of the bacterial community had an obvious positional effect, and there were great differences in the microbial composition in different parts of the community. Endophytes are common in higher plants, and many studies have shown that endophytes can stimulate plant growth or increase natural resistance in host plants. In addition, endophyte and plant cells are closely related and can affect each other’s life activities through physiological metabolism of each other. Therefore, the interaction between endophytes and plant cells is more stable than that of rhizosphere microorganisms. In this study, the fluctuations in the number of endophytic bacteria in all plant lines were seasonal. In June and August, there were no effects on the number of endophytic bacteria between transgenic poplars and non transgenic poplars, whereas there was a greater impact on Endophytic bacteria in October. The variations in endophytic bacteria in the transgenic strains were caused by changes in the season, environment, and plant metabolism, not genetic modifications.

Soil microorganisms are the most sensitive to environmental changes, and soil microbial diversity plays an important role in the structure and function of the soil ecosystem^30,31^. This research showed that transgenic poplars did not exert any influence on the physical and chemical properties of the soil. Moreover, alpha and beta diversity analyses showed that there were no significant differences in bacterial community richness and diversity between transgenic lines and controls, indicating that the transgene did not affect the soil microorganisms. The results of this study were consistent with those of Zhou^32^, who conducted a study on sugarcane containing the *Cry1Ac* gene and found that the transgene did not affect soil microorganisms. Moreover, the distribution of genes from endophytic bacteria and soil microorganisms was obviously positional and seasonal. For example, the number of species in the endophytic bacterial community was lower than that of root and rhizosphere samples. Moreover, bacterial community diversity increased from endophytic soil to rhizoplane soil to rhizosphere soil. Beta diversity analysis also showed that the microbial community structure in rhizoplane and rhizosphere soil were very similar, whereas that of endophytic soil was very different. Hong^33^ studied *Spartina alterniflora* and mangroves and found that the microbial community structure of the root table and rhizospheres were similar, but different from that of endophytic bacteria. These results are consistent with this study. The reason for these results may be because the environmental conditions of the rhizoplane and rhizosphere soil are similar, and the exchange and flow of microorganisms is more frequent and smooth. However, the composition of the endophytic bacteria was affected by plant metabolism and soil environmental changes. Moreover, there were big differences in the metabolic level of different growth stages that affected the composition of the endophytic bacteria. Finally, the composition of endophytic bacteria was different from that found in rhizoplane and rhizosphere samples. The same conclusions were obtained in a study of bacterial communities in transgenic and wild type eucalyptus^34^. There were no significant differences between transgenic and non-transgenic lines, and the changes in the composition of microorganisms in the soil were also seasonal.

In conclusion, a variety of assays showed that the transgenic poplar 741 did not change bacterial diversity in root environments, indicating that there is no risk of gene flow. However, since poplar is a perennial tree, it needs to be monitored for many years in a follow-up study. This study will provide a reference for the ecological security evaluation of transgenic poplars.

## Conclusion

In this study, the *Cry1Ac* and *Cry3A* genes were continuously monitored to identify poplar ecological security issues. This included analyses on gene flow, the arthropod animal community, soil physicochemical properties, and soil microbial diversity. This study found that transgenic poplars could effectively inhibit the number of pests compared with controls. Moreover, transgenic poplar 741 did not affect the stability of the arthropod community. An analysis of the arthropod community showed good ecological effects, and there was no obvious difference between the two groups. For many years, no foreign genes were detected in soil and undergrowth, indicating that gene flow did not occur in the transgenic poplars. The study on microbial diversity showed that the transgenic poplar did not affect the soil physical and chemical properties or the soil microbial community structure. Differences in the microbial community structure were due to location and seasonal effects. The results showed that a 5-year-old transgenic 741 poplar did not pose an ecological risk, and did not affect the microbial community structure and functional diversity. This study provides a reference for the ecological security evaluation of transgenic poplars, and provides a theoretical basis for commercial promotion of transgenic poplars.

## Electronic supplementary material

SUPPLEMENTARY INFO
