# Supplementary material for: A 5-year field study showed no apparent effect of the Bt transgenic 741 poplar on the arthropod community and soil bacterial diversity
Source: Sci Rep. 2018 Jan 31;8:1956. doi: 10.1038/s41598-018-20322-3 (PMC5792429; doi:10.1038/s41598-018-20322-3)
Supplement: Supplementary file 1 — SUPPLEMENTARY INFO [file 41598_2018_20322_MOESM1_ESM.pdf]

Title: A 5-year field study showed no apparent effect of the *Bt* transgenic 741 poplar on the arthropod community and soil bacterial diversity

Authors: Lihui Zuo[1,2¶], Runlei Yang[2,3¶], Zhixian Zhen[1,2], Junxia Liu[1,2], Lisha Huang[4], Minsheng Yang[1,2*]

S1 OUT information for different samples

S2 Correlation of arthropod numbers in different samples

S3 The relative abundance of rhizosphere soil microbial community

S4 Heatmap of 19 phyla bacteria in different samples

S5 Alpha diversity index of different samples

S6 LDA score histogram and evolutionary branches of different samples(Aug_E)

S7 LDA score histogram and evolutionary branches of different samples(Aug_P)

S8 KEGG pathway T-test of different samples(Aug_E)

S9 KEGG pathway T-test of different samples(Aug_P)

S10 KEGG pathway T-test of different samples(Otc_S)

S11 PCR detection of exogenous genes(full-length gels)

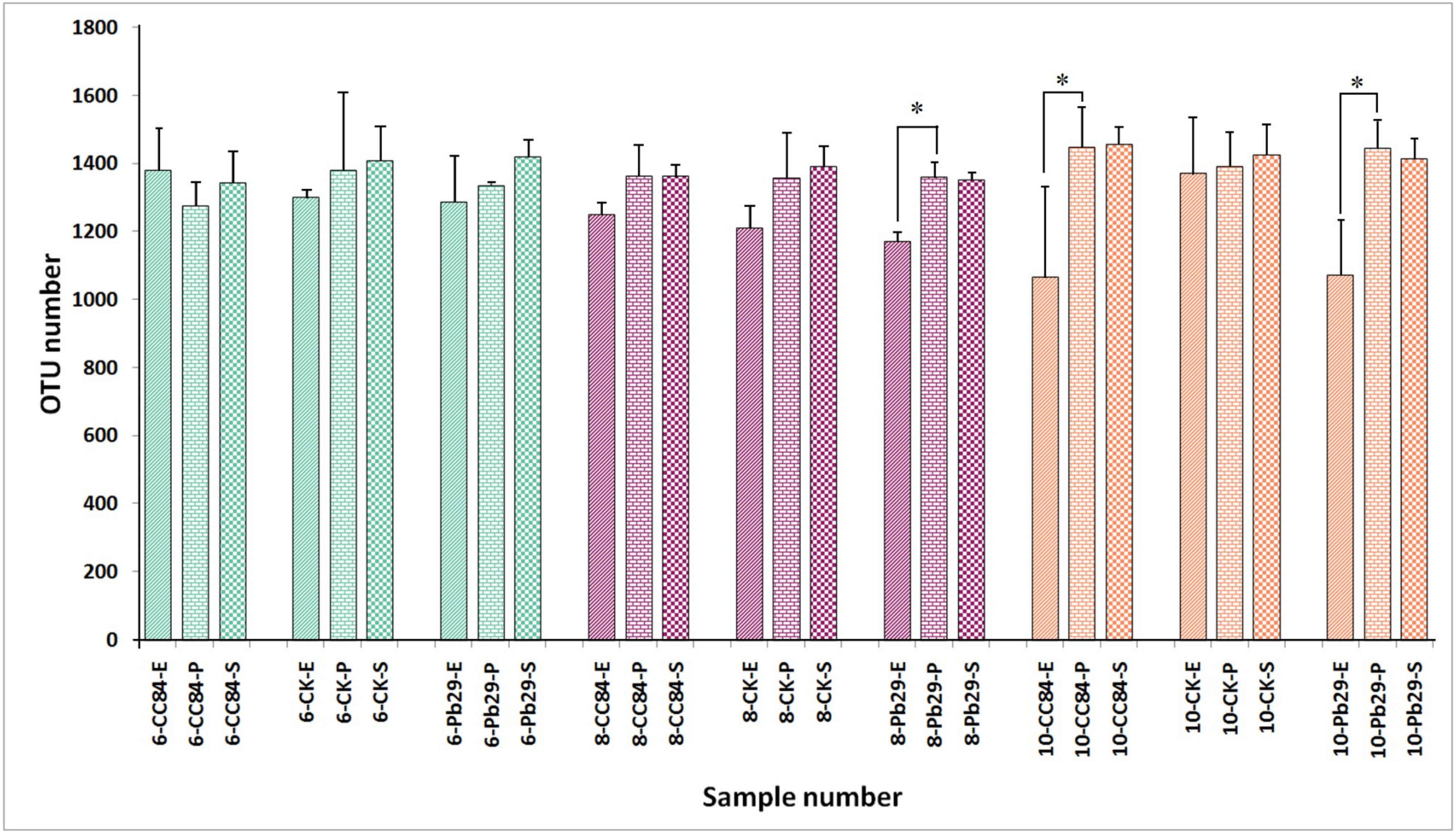

S1 **OUT information for different samples**

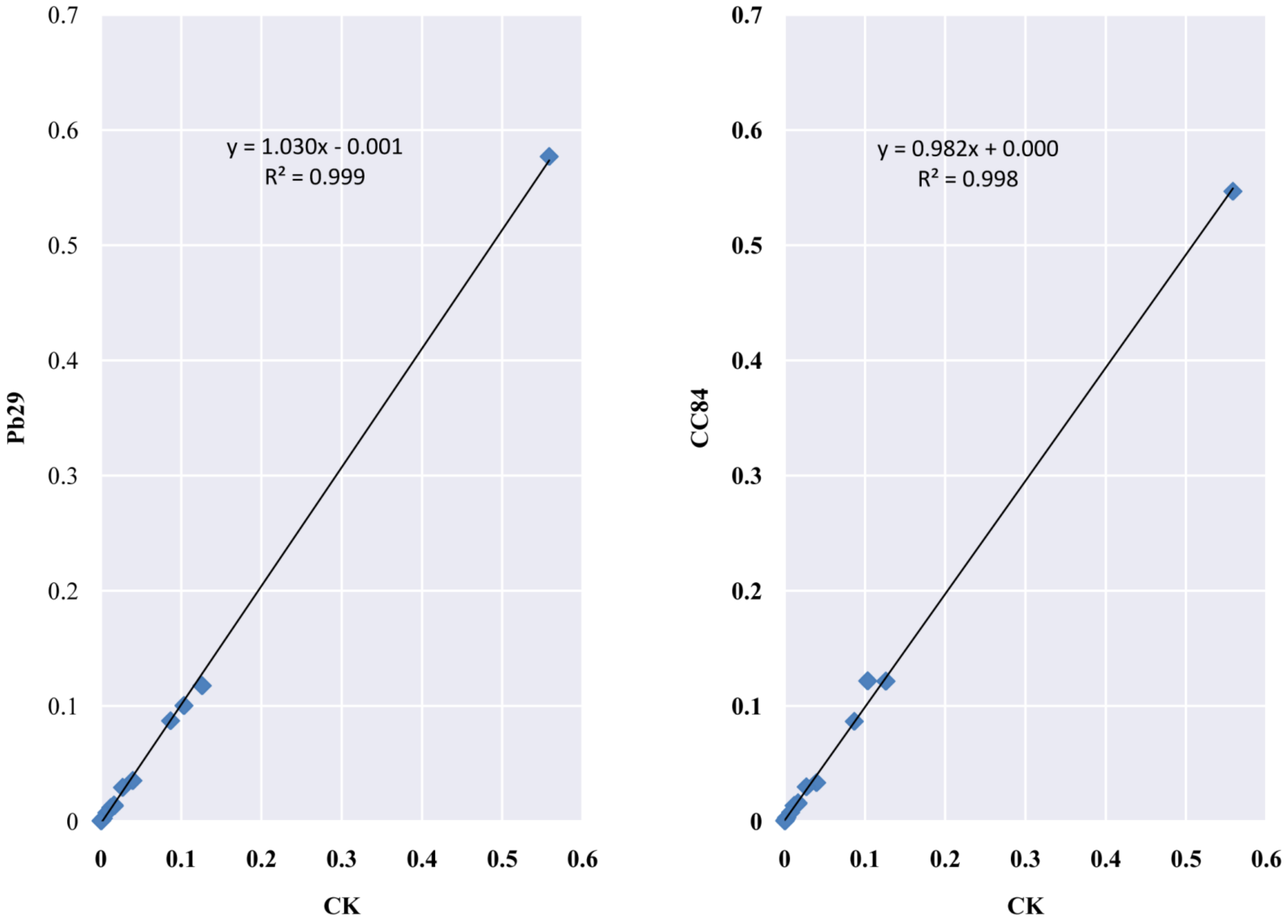

S2 Correlation of arthropod numbers in different samples

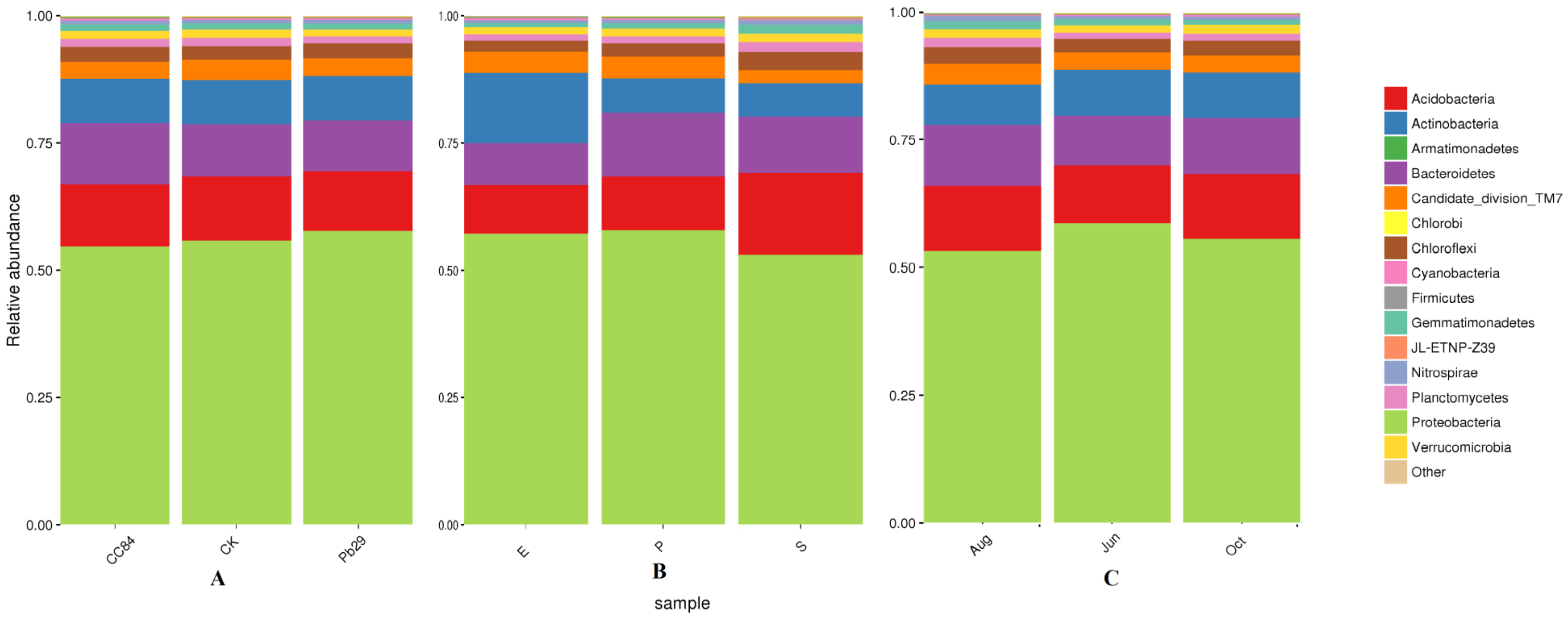

S3 The relative abundance of rhizosphere soil microbial community

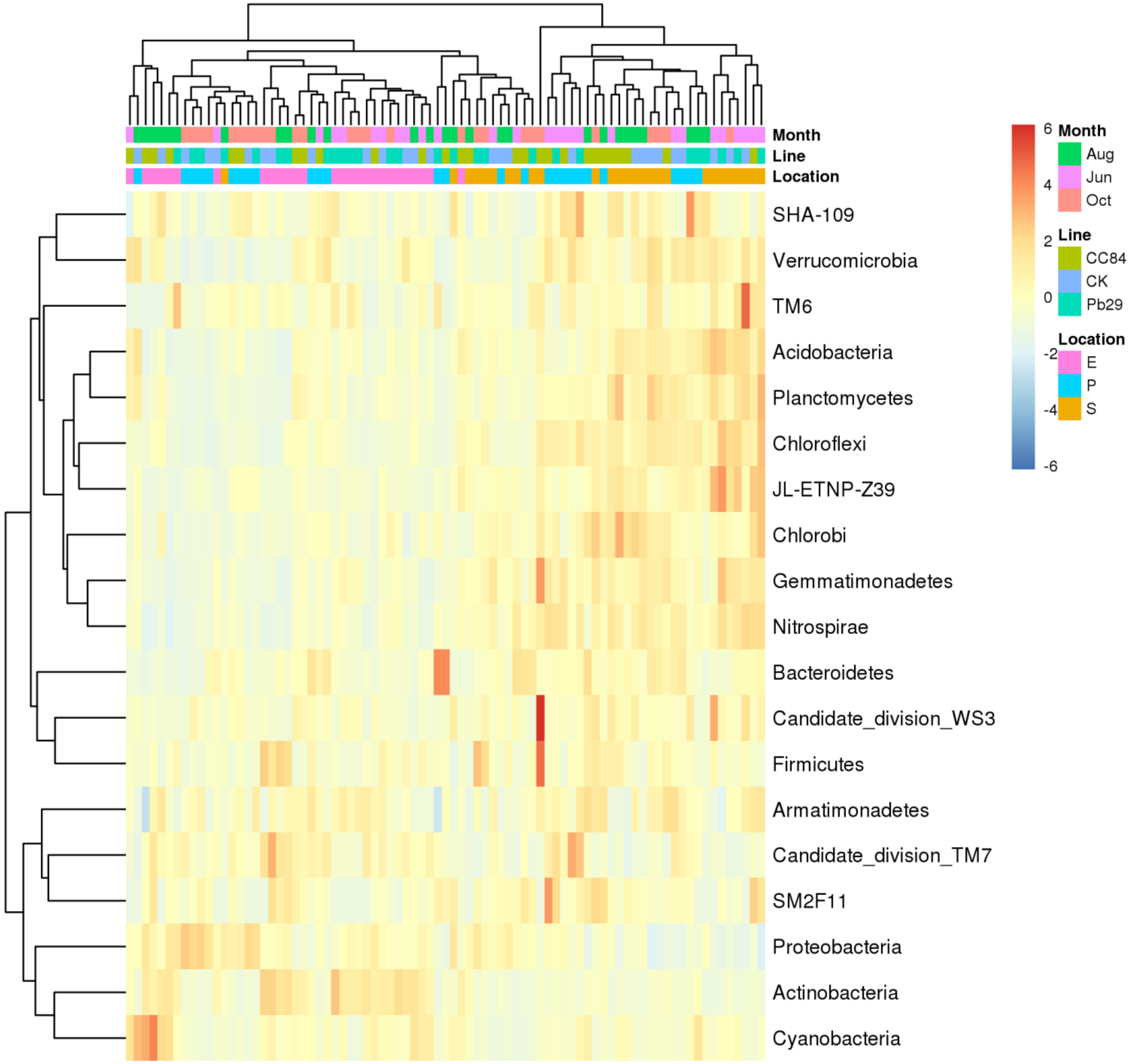

S4 Heatmap of 19 phyla bacteria in different samples

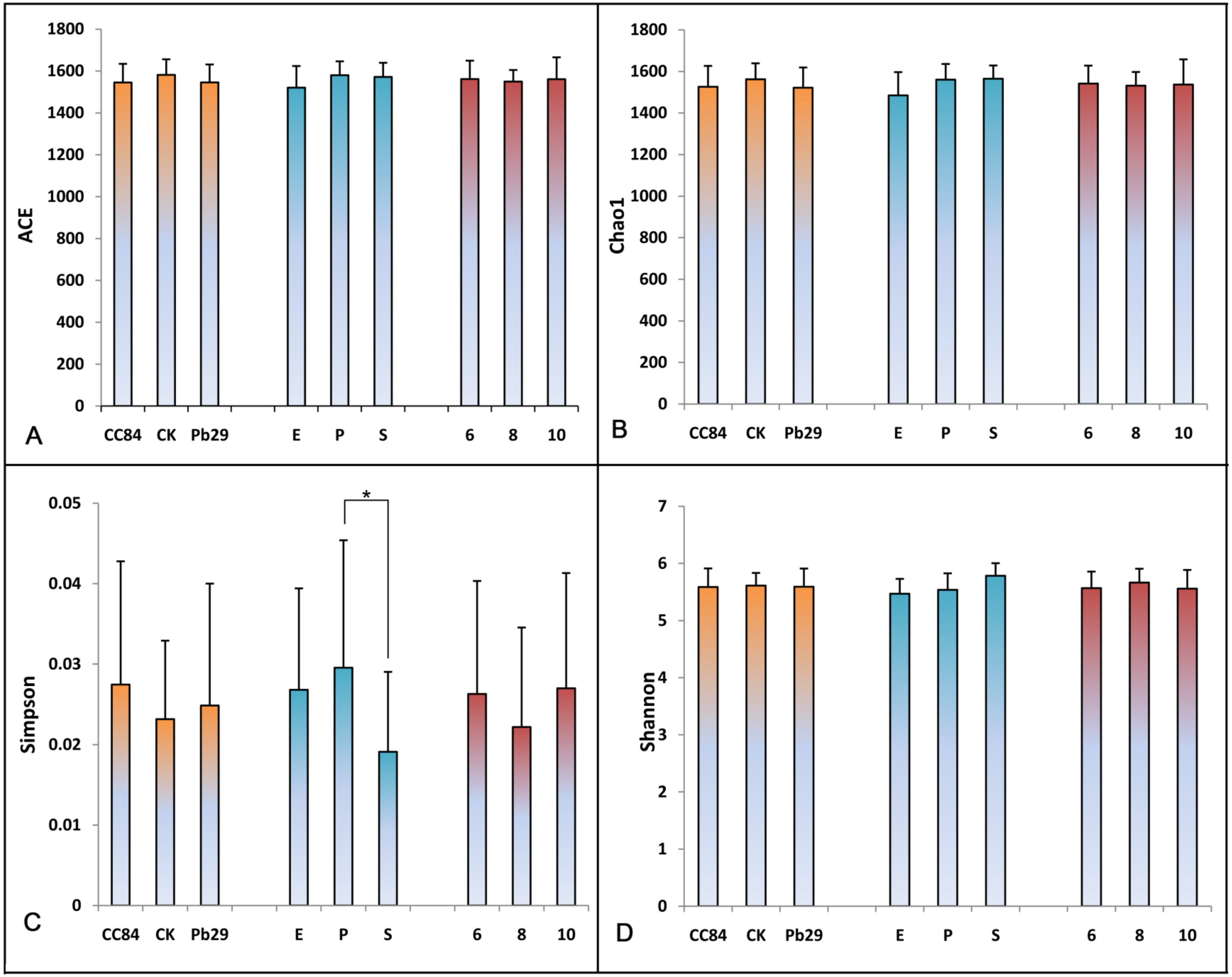

S5 Alpha diversity index of different samples

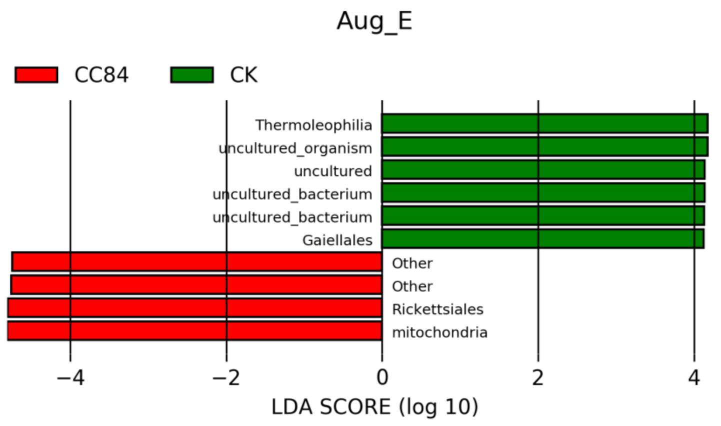

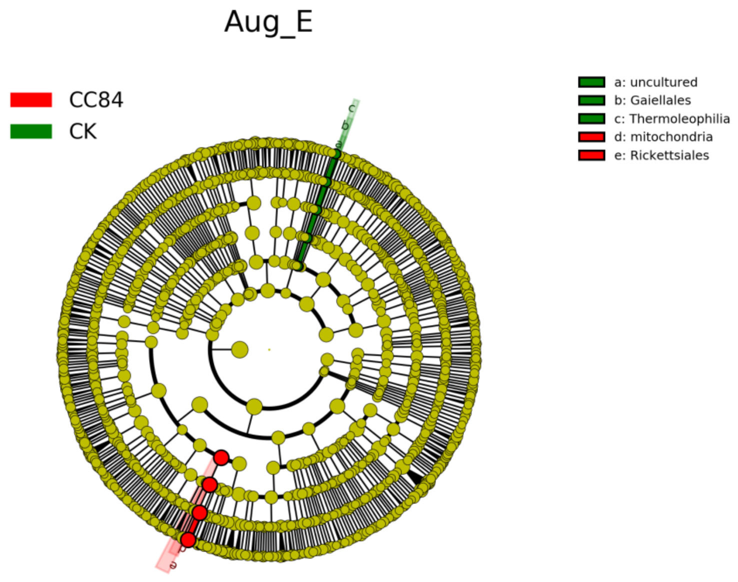

S6 LDA score histogram and evolutionary branches of different samples(Aug_E)

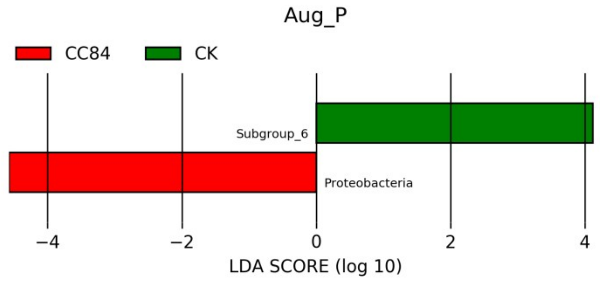

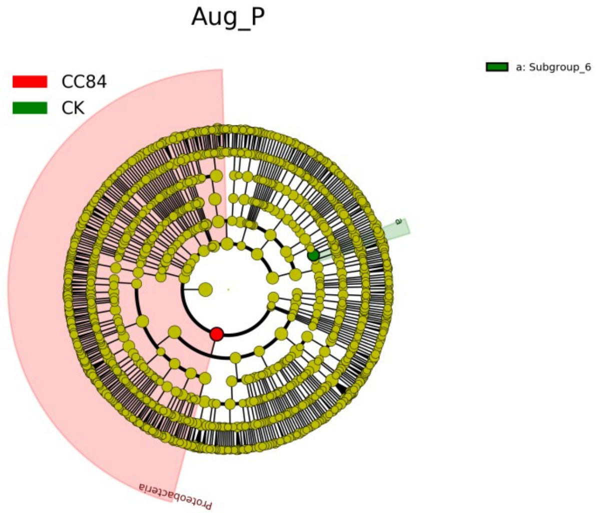

S7 LDA score histogram and evolutionary branches of different samples (Aug_P)

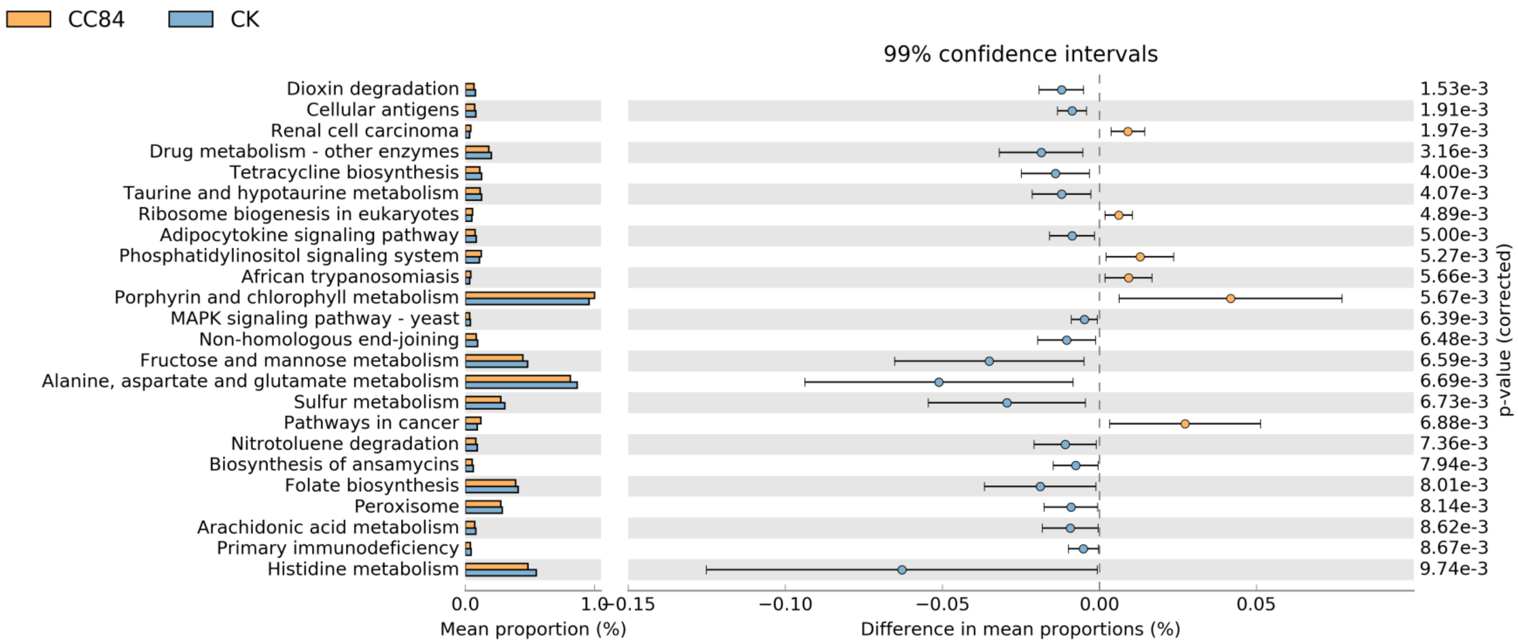

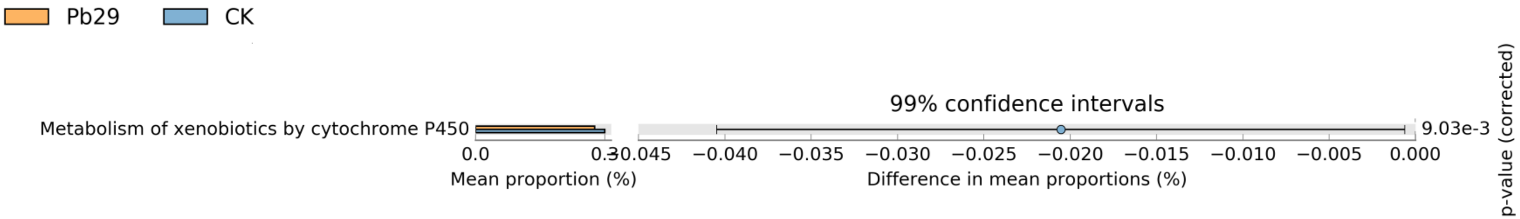

S8 KEGG pathway T-test of different samples(Aug_E)

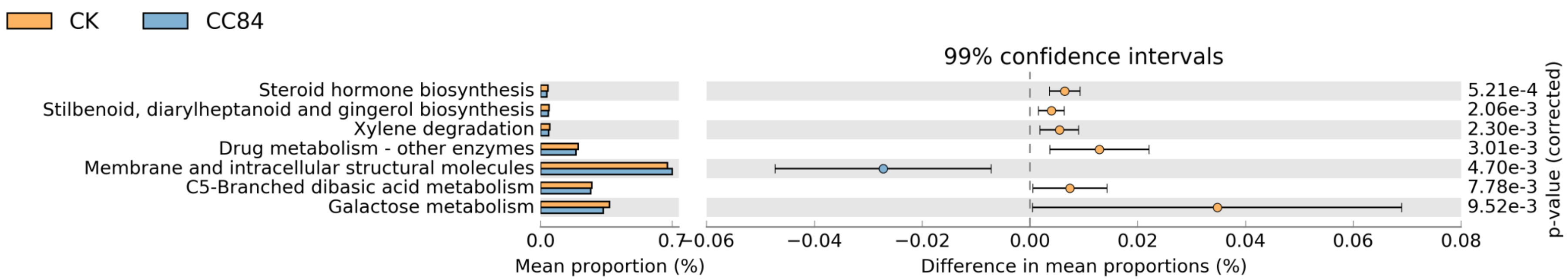

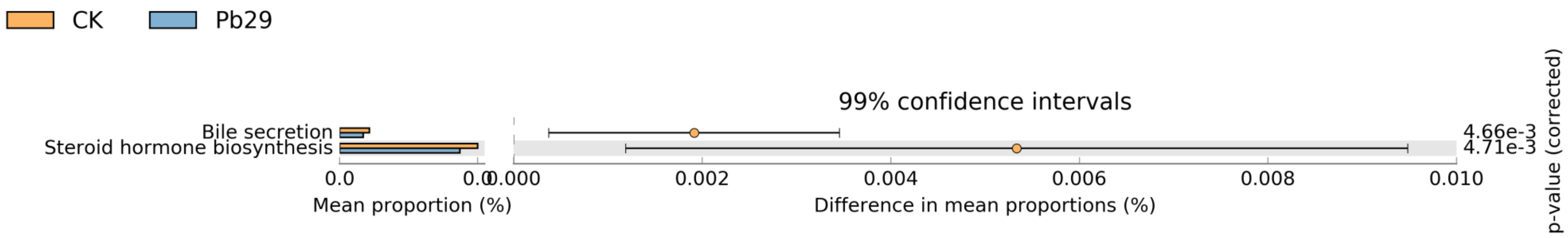

S9 KEGG pathway T-test of different samples(AuG_P)

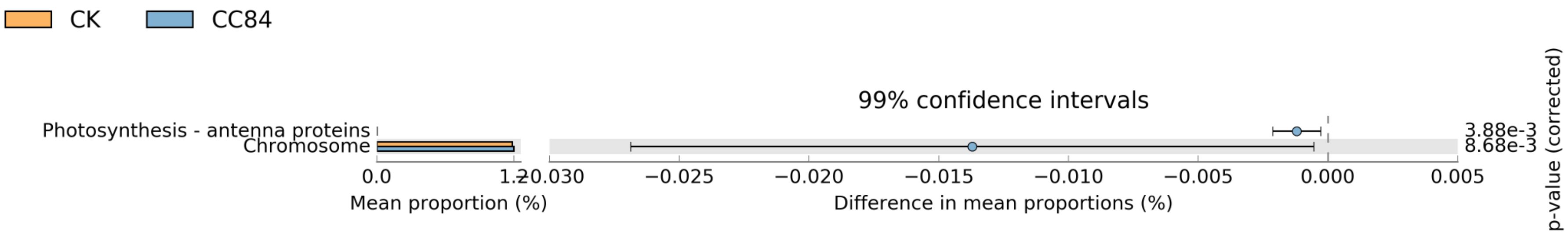

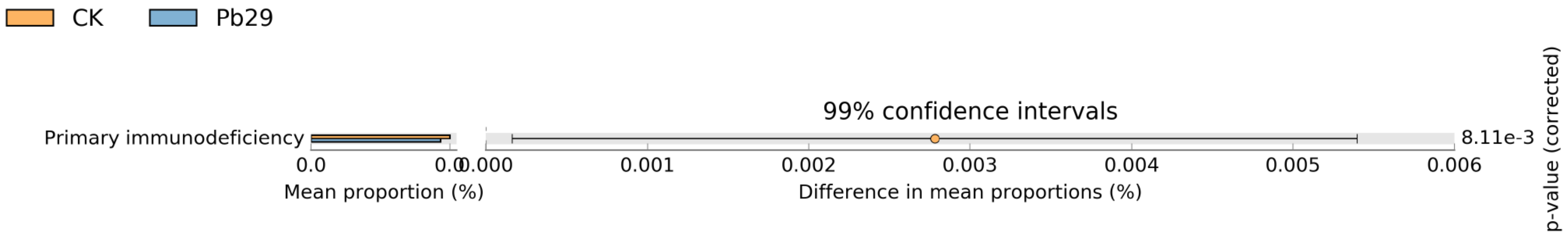

S10 KEGG pathway T-test of different samples(Otc_S)

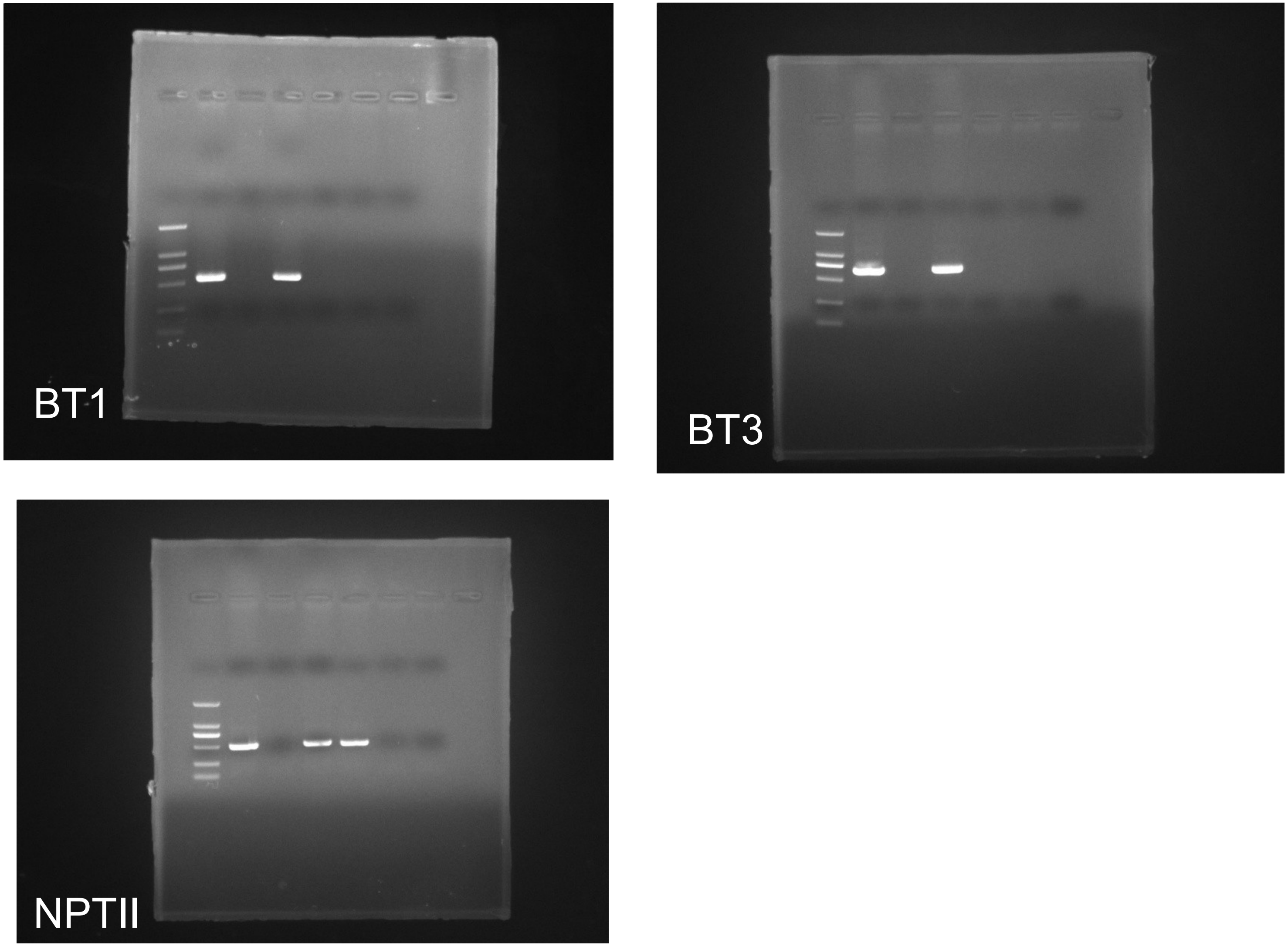

S11 PCR detection of exogenous genes(full-length gels)
